# Anti-Angiogenic and Anti-Inflammatory Properties of Kahweol, a Coffee Diterpene

**DOI:** 10.1371/journal.pone.0023407

**Published:** 2011-08-09

**Authors:** Casimiro Cárdenas, Ana R. Quesada, Miguel A. Medina

**Affiliations:** 1 Department of Molecular Biology and Biochemistry, Faculty of Sciences, University of Málaga, Málaga, Spain; 2 CIBER de Enfermedades Raras (CIBERER), Málaga, Spain; IIT Research Institute, United States of America

## Abstract

**Background:**

Epidemiological studies have shown that unfiltered coffee consumption is associated with a low incidence of cancer. This study aims to identify the effects of kahweol, an antioxidant diterpene contained in unfiltered coffee, on angiogenesis and key inflammatory molecules.

**Methodology/Principal Findings:**

The experimental procedures included *in vivo* angiogenesis assays (both the chicken and quail choriallantoic membrane assay and the angiogenesis assay with fluorescent zebrafish), the *ex vivo* mouse aortic ring assay and the *in vitro* analysis of the effects of treatment of human endothelial cells with kahweol in cell growth, cell viability, cell migration and zymographic assays, as well as the tube formation assay on Matrigel. Additionally, two inflammation markers were determined, namely, the expression levels of cyclooxygenase 2 and the levels of secreted monocyte chemoattractant protein-1. We show for the first time that kahweol is an anti-angiogenic compound with inhibitory effects in two *in vivo* and one *ex vivo* angiogenesis models, with effects on specific steps of the angiogenic process: endothelial cell proliferation, migration, invasion and tube formation on Matrigel. We also demonstrate the inhibitory effect of kahweol on the endothelial cell potential to remodel extracellular matrix by targeting two key molecules involved in the process, MMP-2 and uPA. Finally, the anti-inflammatory potential of this compound is demonstrated by its inhibition of both COX-2 expression and MCP-1 secretion in endothelial cells.

**Conclusion/Significance:**

Taken together, our data indicate that, indeed, kahweol behaves as an anti-inflammatory and anti-angiogenic compound with potential use in antitumoral therapies. These data may contribute to the explanation of the reported antitumoral effects of kahweol, including the recent epidemiological meta-analysis showing that drinking coffee could decrease the risk of certain cancers.

## Introduction

**Figure 1:**
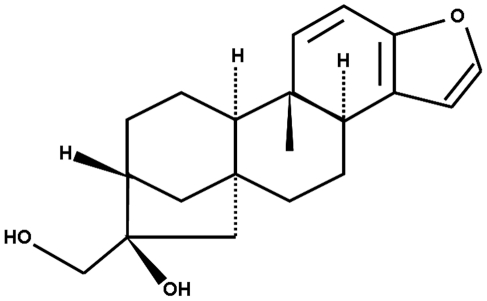
Chemical structure of kahweol.

Many different foods contain non-nutritional components that can have beneficial effects to the health [1]. This is the case of coffee, which includes more than a thousand of compounds [2]. One of these is kahweol (Figure 1), an antioxidant diterpene that remains in unfiltered coffee beverages, such as Turkish and Scandinavian coffee [3]. Epidemiological studies associate the consumption of unfiltered coffee with a low incidence of colon and liver cancer [4], [5]. Furthermore, its preventive effects against oxidative stress and DNA damage are well described [6].

Angiogenesis is a hallmark of cancer, required for both cancer progression and metastasis [7]. Mechanistically, angiogenesis is a very complex process in which several key steps are involved [8]. In fact, when quiescent endothelial cells are activated by some proangiogenic signal, they change their phenotype to become highly proliferative and able to migrate, remodel the surrounding extracellular matrix (ECM) and finally to differentiate to form new vessels. Any of these key steps can be a potential pharmacological target to inhibit angiogenesis and, hence, to treat angiogenesis-dependent diseases [9].

Our group is actively involved in the search for new modulators of angiogenesis from natural sources [10], [11], [12], [13], [14]. In the present study, the effects of kahweol on two *in vivo* and one *ex vivo* model of angiogenesis and on several key steps of the process are described. They include its effects on endothelial cell “differentiation” to yield tubular-like structures, endothelial and tumor cell proliferation, apoptosis, and migration, as well as its effects on extracellular matrix remodelling enzyme activities of matrix metalloproteinase-2 (MMP-2) and urokinase-type plasminogen activator (uPA). We have also investigated kahweol antiinflammatory potential through cyclooxygenase-2 (COX-2) and monocyte chemoattractant protein 1 (MCP-1) modulation. Our results reinforce the potential pharmacological interest of kahweol, as suggested by its anti-angiogenic and anti-inflammatory effects.

## Results

### Kahweol inhibits in vivo angiogenesis

**Figure 2:**
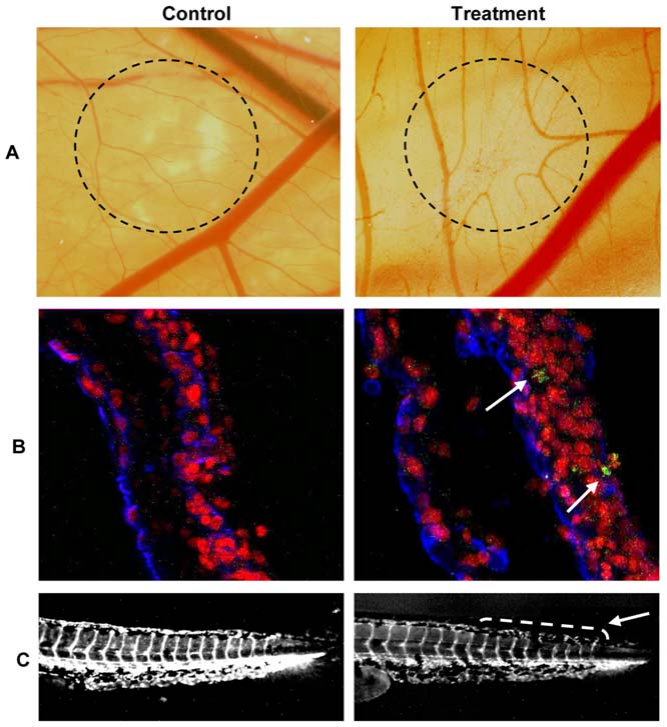
Kahweol inhibits *in vivo* angiogenesis and does not induce endothelial cell-specific apoptosis in the quail CAM assay. A) CAM assay. Dotted circles identify the position of the methyl cellulose discs after incubation, carried out as described in Materials and methods. In controls, methyl cellulose discs were prepared with the vehicle (DMSO). In treatments, methyl cellulose discs contained 50 nmol of kahweol. B) Detection of apoptosis in the quail CAM assay. Arrows point to apoptotic cells. C) Angiogenesis assay in the zebrafish model. The arrow points to the caudal region with narrower and disrupted intersegmental vessels in kahweol treated zebrafish embryos.

The chicken chorioallantoic membrane (CAM) assay was used to determine the ability of kahweol to inhibit angiogenesis *in vivo*. Although 50 nmol of kahweol per CAM was required to observe *in vivo* inhibition of angiogenesis in 100% of treated eggs, as little as 10 nmol of kahweol was enough to induce clear inhibition of angiogenesis in 25% of the tested eggs in the CAM assay. Figure 2 (upper lane) shows that kahweol treatment induced disorganization and inhibition of the ingrowth of new vessels in the area covered by the discs containing the compound. It can also be observed that the peripheral vessels (relative to the position of the disc) grew centrifugally, avoiding the treated area, with an overall decrease in the vascular density.

A second experimental approach used to test the effects of kahweol on *in vivo* angiogenesis was the use of a model of transgenic zebrafish. Figure 2 (lower line) shows representative images of the effects of 75 µM kahweol for 24 h on intersegmental vessels of 3 days-post mating larvae. The results obtained showed a decrease in the width of some vessels and interruptions in other vessels. A quantitative analysis of these effects revealed that 85% of 75 µM kahweol-treated and 75% of 25 µM kahweol-treated larvae exhibited inhibited angiogenesis (results not shown). This inhibitory effect is unambiguously shown by video recording of blood flow. In the supplementary material, Video S1 shows clearly a continuous blood flow along intersegmentals vessel of control zebrafish larvae. In contrast, Video S2 shows that larvae treated with 50 µM kahweol for 24 h exhibited no blood flow along intersegmental vessels.

### Kahweol induces non-specific cell apoptosis in quail CAM

A modified CAM assay in the quail has been adopted in our laboratory, which makes possible to stain simultaneously endothelial cells and apoptotic nuclei [15]. Figure 2 (middle line) shows that 50 nmol kahweol induced apoptosis in a small percentage of cells but this effect did not seem to be endothelial cell specific, since only few apoptotic nuclei corresponded to endothelial cells.

### Kahweol inhibits endothelial cell sprouting in the mouse aortic ring assay

**Figure 3:**
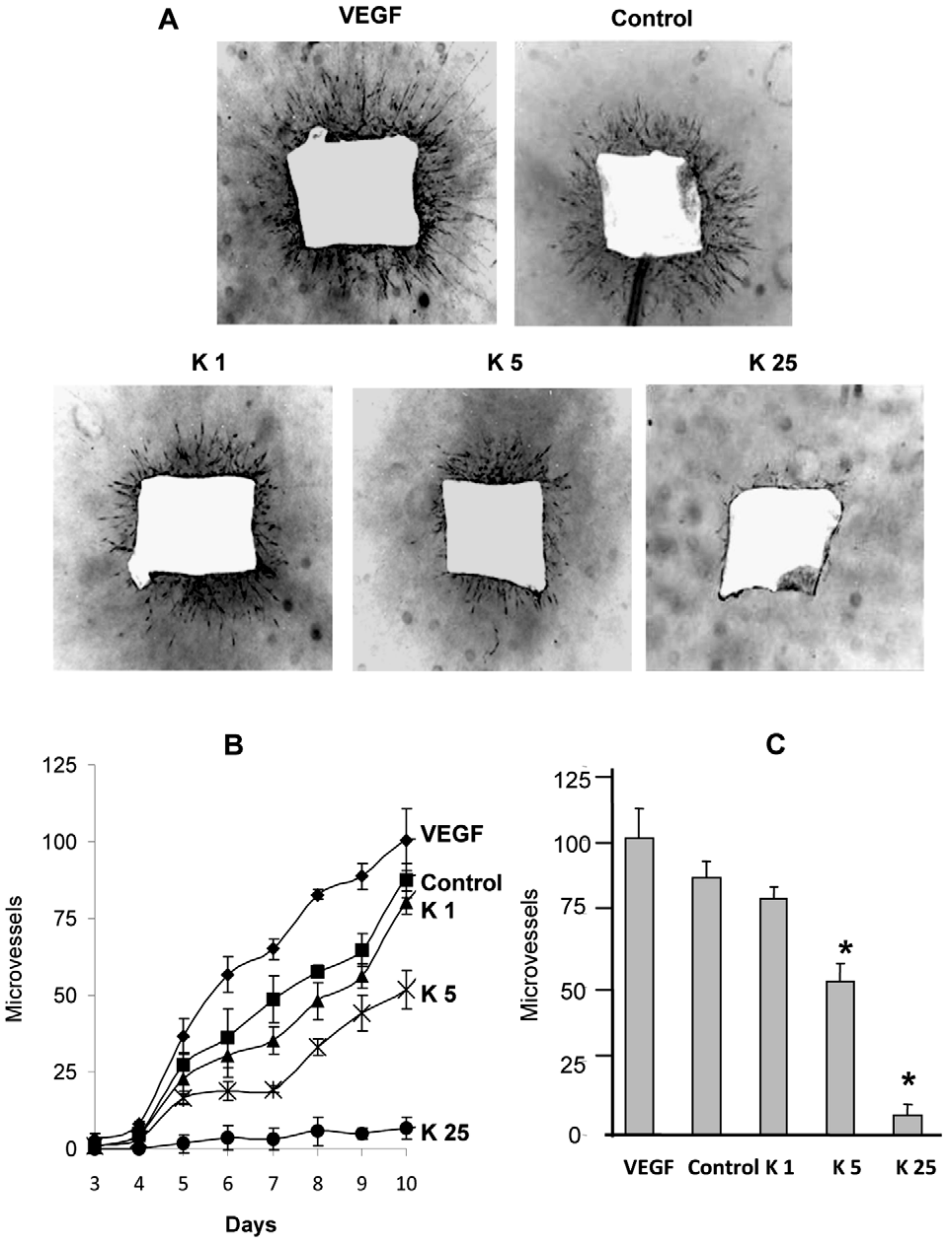
Kahweol inhibits endothelial cell sprouting from aortic rings in a dose-dependent manner. Aortic ring assay was performed as described in Materials and methods. A) Negative of photographs (x20) of aortic rings (lateral view) after 10 days of incubation in a 3D collagen gel overlayed with complete medium in the presence of 20 mg/mL VEGF, 0.05% DMSO (the vehicle taken as a control), or kahweol at 1, 5 and 25 µM (K1, K5, K25, respectively). Experiments were repeated at least three times. B) Microvessel time course for the different treatments mentioned in A. Data are given as microvessel total count at different incubation times (spanning from 3 to to 10 days), and they are means±S.D. of three different experiments. C) Microvessel total count after 10 days of incubation. Data are given as microvessel total count, and they are means±S.D. of three different experiments. *Statistically significant (p<0.01) as compared to control values, according to a two-tailed Student's *t-*test.

A third line of evidence showing the potential of kahweol to inhibit overall angiogenesis is provided by the *ex vivo* model of the mouse aortic ring assay. Figure 3 shows that under control conditions the aortic ring was able to generate new vessel sproutings and that the density of these sproutings increased in the presence of the proangiogenic agent VEGF. As Figure 3 shows, kahweol treatment inhibited endothelial cell sprouting (panel A), and this effect was observed along treatment from day 5 to 10 (panel B). Furthermore, a clear dose-response inhibition of microvessel formation was observed and quantified in treatments with kahweol. In fact, 5 µM kahweol was able to inhibit microvessel sprouting by 40%, whereas 25 µM kahweol almost completely inhibited this angiogenic response after 10 days of treatment (panel C).

### Kahweol inhibits endothelial cell proliferation

Angiogenesis involves local proliferation of endothelial cells in response to an angiogenic stimulus. However, the desirable endothelial cell specificity of this effect is not a common feature [11].

**Figure 4:**
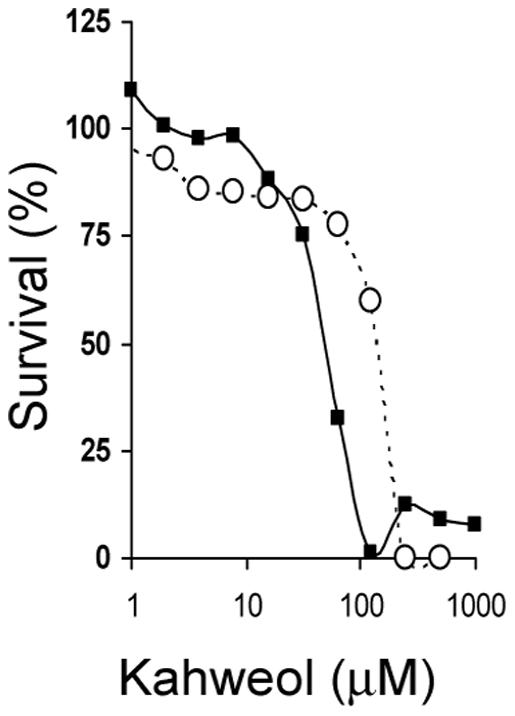
Kahweol inhibits endothelial cell proliferation. Survival curves of proliferative (squares) and non-proliferative (circles) HUVEC endothelial cells treated with kahweol. Concentrations are represented in logarithmic scale. Depicted data are means of values of three independent experiments (each one with quadruplicate samples). Standard deviation values (in all the cases lower than 20% of mean values) are not represented for the sake of clarity.

Therefore, we studied the effects of kahweol on the growth of endothelial cells. Figure 4 shows the mean survival curves obtained with the MTT assay in human umbilical vein endothelial cells (HUVEC), under conditions of normal and low proliferation rates (20% and 2% of FBS). From these curves, estimated IC_50_ values were 50±1 and 147±7 µM for proliferative and non-proliferative HUVEC cells, respectively. This effect on cell survival was not endothelial cell-specific, since IC_50_ values for kahweol treatment of several human tumoral cell lines were similar to those obtained for HUVEC (results not shown).

### Kahweol does not induce apoptosis on HUVEC

Cell growth is the result of the balance between their proliferation and death rates. Therefore, it would be advisable to test the potential effects of kahweol on endothelial cell apoptosis. However, a treatment with 25 µM kahweol was not able to induce apoptosis in HUVEC (results not shown). As a positive control, a treatment with 10 µM 2-methoxyestradiol was used.

### Kahweol inhibits tubule formation of endothelial cells on Matrigel

**Figure 5:**
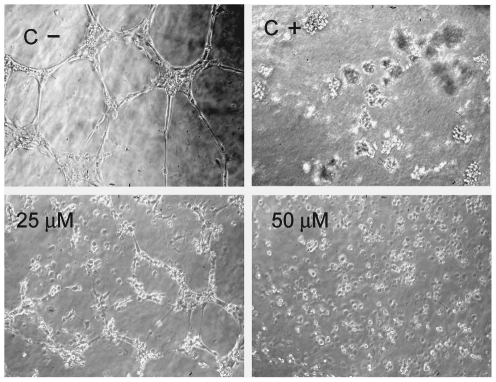
Kahweol inhibits tubule formation of endothelial cells on Matrigel in a dose-dependent manner. Data are representative of, at least, three independent experiments.(C−) Negative controls, HUVEC on Matrigel with no treatment. (C +) Positive controls, HUVEC on Matrigel treated with 50 µM suramin.

The final event during angiogenesis is the formation of a three-dimensional network of tubes by endothelial cells. *In vitro*, endothelial cells plated on Matrigel align themselves forming cords (Figure 5, C −, negative controls). Figure 5 shows the effect of two different concentrations of kahweol in this assay after 6 h of treatment. As a positive control for total inhibition, 50 µM suramin-treatment for 6 h was used (Figure 5, C+). The kahweol concentrations required to inhibit the differentiation of HUVEC cells did not affect their viability (results not shown).

### Kahweol inhibits HUVEC endothelial cell migration

**Figure 6:**
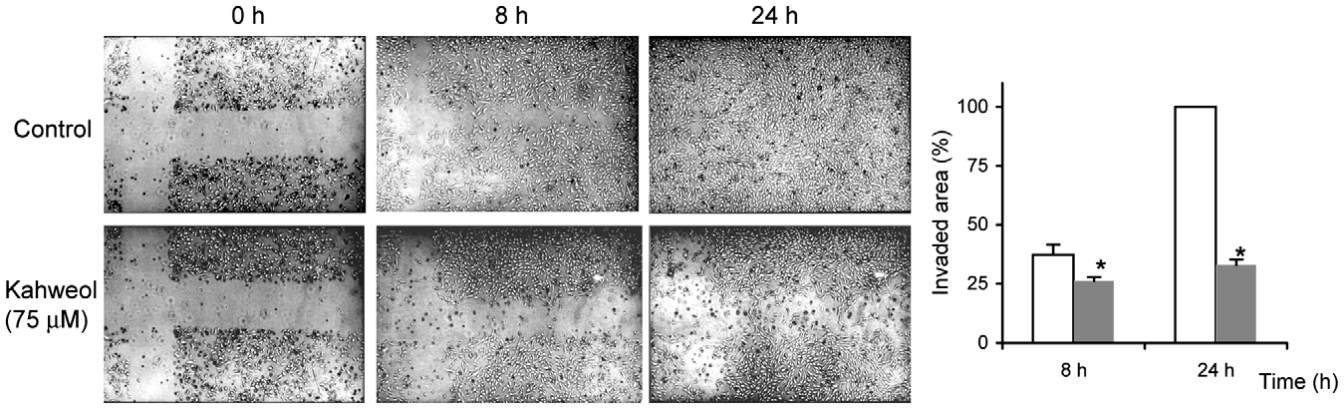
Kahweol inhibits endothelial cell migration. Photographs were taken on untreated (control) and 75 µM kahweol-treated HUVEC cells at 0, 8 and 24 h after “wounding”. Data are representative of, at least, three independent experiments. At the right, the counting of HUVEC migration into the “wounded” area at 8 and 24 h after “wounding” is depicted. Data are given as percentages of re-occupied “wounded” area and they are means±S.D. of three different experiments. White bars are control values and grey bars correspond to treatments. *Statistically significant (p<0.01) as compared to control values, according to a two-tailed Student's *t-*test.

Cell migration is a key step shared by both angiogenesis and tumor progression. Figure 6 shows the effects of 75 µM kahweol on endothelial cell migration, as determined by the “wound healing” assay, after 8 and 24 h of treatment. Quantitative determination of the invaded area shows a significant 30 and 66% inhibitory effect of kahweol after 8 and 24 h of treatment, respectively.

### Kahweol inhibits endothelial cell invasion

**Figure 7:**
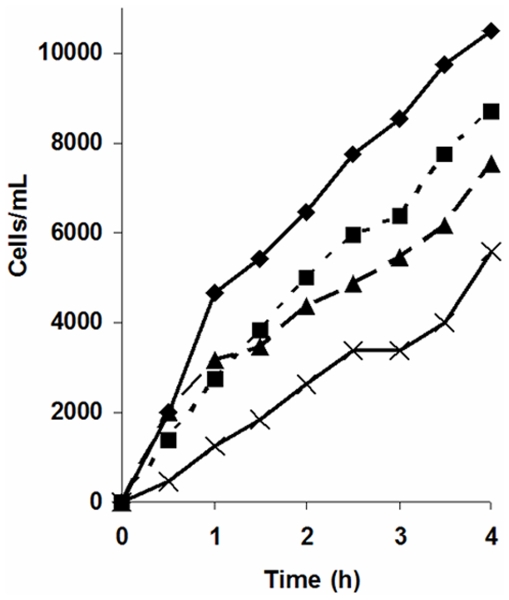
Kahweol inhibits HUVEC endothelial cell invasion in a dose-dependent manner. Invading controls and 25 and 75 µM kahweol-treated HUVEC cell count values are represented by using diamonds, squares and triangles, respectively. As negative controls, the number of untreated invading HUVEC to wells not containing chemoattractant was determined (crosses). Data are given as number of invading cells and they are means of two different assays (each one carried out in triplicate).

Cell invasion is another key step of angiogenesis. Data obtained on the effects of kahweol on endothelial cell invasion (as determined by a continuous fluorescent assay) clearly show that kahweol induces an anti-invasive effect in HUVEC in a dose-dependent manner (Figure 7). In fact, the rate of invasion was inhibited a 23, 33 and 52% (as compared to the rate of invasion for control, untreated cells) by 25, 50 and 75 µM kahweol treatments, respectively.

### Kahweol inhibits endothelial cell MMP-2 and urokinase

MMP-2 and uPA are extracellular matrix remodeling ezymes expressed by endothelial cells and involved in angiogenesis.

**Figure 8:**
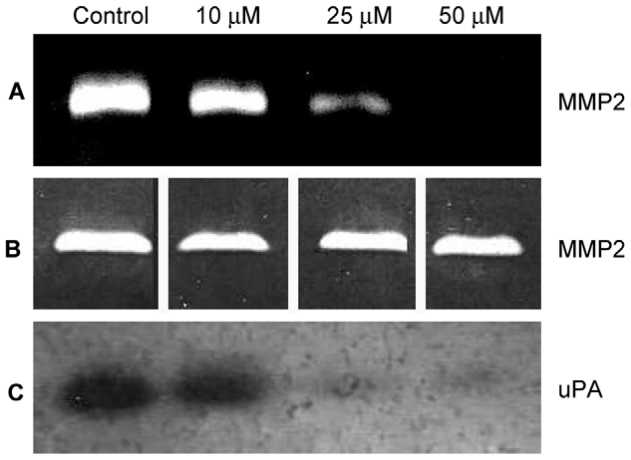
Kahweol inhibits HUVEC MMP-2 and uPA in a dose dependent manner. A) Gelatin zymography of MMP-2 in conditioned media of HUVEC after treatment with different kahweol concentrations. B) *In situ* determination of kahweol effects on HT-1080 gelatinases, as determined by gelatin zymography with the presence of kahweol in the incubation substrate buffer. C) Plasminogen zymography of HUVEC uPA after treatment with different kahweol concentrations.

Figure 8 (A) shows that kahweol inhibits HUVEC MMP-2 expression, with a clear dose response effect and complete inhibition by 50 µM kahweol. An *in situ* activity assay with HT-1080 gelatinases (Figure 8 B) shows that this is not a direct effect on the enzyme.

Figure 8 (C) shows that kahweol-treatment induces a dose-dependent decrease in the levels of urokinase in HUVEC conditioned media, with an almost complete inhibition at 50 µM kahweol.

### Kahweol inhibits endothelial cell COX-2 expression and MCP-1 secretion

**Figure 9:**
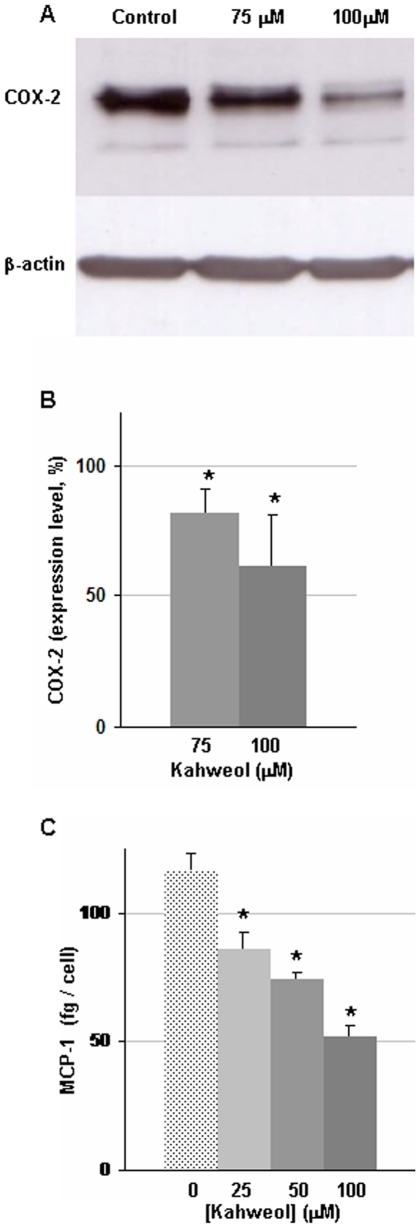
Kahweol inhibits HUVEC endothelial cell COX-2 expression and MCP-1 secretion in a dose-dependent manner. A) Typical results of a Western blot assay using anti-COX-2 antibodies. B) Quantification of the normalized relative inhibitory effect. Data are given as percentage, taking the normalized levels of COX-2 in control cells as 100%, and they are means±S.D. of three different assays. C) Quantification of the amount of MCP-1 secreted by HUVEC after a 24 h treatment in the presence of different concentrations of kahweol. Data are given as femtograms of secreted MCP-1 per cell, and they are means±S.D. of three different experiments. *Statistically significant (p<0.01) as compared to control values, according to a two-tailed Student's *t-*test.

COX-2 is an important pro-inflammatory protein expressed at high levels in tumoral angiogenic vessels. Figure 9 (A and B) shows that kahweol inhibits in a dose dependent manner the expression of COX-2 protein by HUVEC.

Monocyte chemoattractant protein-1 (MCP-1) is a key protein mediating inflammatory processes. Endothelial cells do express and secrete MCP-1. Figure 9 (C) shows that kahweol treatment also induced a dose-dependent inhibition on MCP-1 secretion by HUVEC.

## Discussion

Angiogenesis plays a key role in tumor growth, invasion and metastasis. However, the results obtained in the clinical treatment of cancer with approved antiangiogenic compounds show only limited -although significant- improvement [16], [17]. It should be stressed that this first generation of antiangiogenic compounds targets the first step of VEGF biosignaling. As we have previously suggested, since tumor angiogenesis is very complex and involves a number of different cell types, a multi-target approach for the anti-angiogenic treatment of cancer could be expected to produce better results [18]. Therefore, new multi-targeted compounds (or combinations of them) are urgently required to be introduced in the clinical setup. The results shown in the present study clearly indicate that kahweol is another natural anti-angiogenic compound with a wide spectrum of targets [10], [11], [12], [19], [20].

In the CAM assay, the inhibitory doses exhibited by kahweol are similar to those of other anti-angiogenic compounds found by us to inhibit angiogenesis in the CAM assay [10], [19], and much lower than those of other anti-angiogenic compounds [21]. The global morphological features (including centrifugal growth of the peripheral vessels -relative to the position of the disc-, avoiding the treated area, with an overall decrease in the vascular density) elicited by kahweol treatment are also in agreement with those previously observed for other anti-angiogenic compounds. On the other hand, the present research work shows a confirmatory evidence of the potential of kahweol to inhibit *in vivo* angiogenesis, by using another completely independent model system, namely, that of genetically modified zebrafish. Furthermore, a third independent approach (the mouse aortic ring assay) confirms the high potential of kahweol to inhibit angiogenesis in a nice dose-dependent manner.

The CAM and zebrafish *in vivo* assays and the *ex vivo* mouse aortic ring assay clearly identify kahweol as a new anti-angiogenic compound, but gives no information on which specific steps of angiogenesis are targeted by this compound. To characterize an anti-angiogenic compound, it is advisable to study its effects on the different steps involved in angiogenesis. To get new, additional insights on the features of kahweol as an anti-angiogenic compound, we carried out a complete set of *in vitro* assays previously used by us to characterize the anti-angiogenic effects of other compounds from natural sources, including aeroplysinin-1, homocysteine, ursolic acid, puupehenone, hypericin, hyperforin and aloe-emodin, among others [11], [12], [13], [19], [20], [21].

Angiogenesis involves local proliferation of endothelial cells in response to an angiogenic stimulus. In fact, several of the best characterized anti-angiogenic compounds were initially detected and selected for their capability to interfere with endothelial cell growth. This is the case of the extremely selective inhibitor of endothelial cell proliferation TNP-470, a synthetic analog of fumagillin with enhanced anti-angiogenic properties [22], [23]. Many other natural anti-angiogenic compounds inhibit endothelial cell proliferation [9], [10], [24], [25]. However, the desirable endothelial cell specificity of this effect is not a common feature [11]. Our data obtained with the MTT assay suggest a non-specific cytotoxic effect of long term (3 days) treatments of both endothelial and tumor cells with micromolar concentrations of kahweol. Hence, kahweol seems to behave not only as a potential anti-angiogenic compound but also as a potential anti-tumoral compound, in agreement with previous observation from other groups [26]. On the other hand, IC_50_ value for non-proliferative HUVEC was 3-fold that obtained for proliferative HUVEC, as expected.

Concerning the apoptosis assay, the negative effect on endothelial (HUVEC) cells is in agreement with our results in quail CAM. On the other hand, these data suggest that the potential effects of kahweol on apoptosis could exhibit certain cell specificity. Future studies in a wider range of tumor and endothelial cell types seem warranted.

The final event during angiogenesis is the organization of endothelial cells in a 3-D network of tubes. *In vitro*, endothelial cells plated on Matrigel align themselves forming cords, already evident a few hours after plating. The minimal inhibitory concentration for kahweol in this assay of “tubule-like” structures formation on Matrigel was 25 µM, in the range of concentrations at which other known antiangiogenic compounds produce this kind of effect [13], [27]. Therefore, kahweol-treatment has another key target in this essential step of the angiogenesis process.

Migration of endothelial cells is required for angiogenesis to proceed. The results obtained in the “wound healing” assay clearly show that 75 µM kahweol is able to inhibit endothelial cell migration.

A key feature of endothelial cells switched to their angiogenic phenotype is their ability to invade the surrounding space. Our data also show that invasion is inhibited by kahweol. Since invasion is dependent on extracellular matrix remodeling capabilities, this inhibitory effect strongly suggested that the two key extracellular membrane remodeling enzymes expressed by endothelial cells, namely, MMP-2 and uPA could be other main key targets of the pharmacological action of kahweol on endothelial cells. Both proteases play key roles in angiogenesis, being involved in the positive proteolytic balance required for capillary sprout elongation and lumen formation during angiogenesis [28], [29], [30], [31]. Matrix metalloproteinases 2 and 9 (MMP-2, MMP-9), commonly named gelatinases, are two key extracellular enzymes involved in ECM remodeling, which is an essential step required not only for angiogenesis, but also for metastasis [32], [33], [34]. uPA is a serine protease that is also involved in ECM remodeling related to angiogenesis and metastasis [35], [36], [37]. Our results in the zymographic assays for gelatinase and urokinase activities clearly showed that, in fact, kahweol was able to inhibit the expression of both MMP-2 and uPA, identifying them as two relevant molecular targets for kahweol.

On the other hand, the anti-oxidant nature of kahweol also points to its potential anti-inflammatory capabilities. Our results indicate that kahweol inhibits two key inflammatory mediators, COX-2 and MCP-1, which are also related with angiogenesis. It has been shown previously that kahweol exerts a suppressive effect on COX-2 expression in macrophages [38]. Recent studies have shown that COX-2 and MCP-1 receptor (CCR-2) induction in HUVEC is related to increased levels of VEGF and that specific antagonists of CCR2 decrease VEGF levels [39]. Furthermore, in COX-2 deficient mice a decrease in VEGF, and both reduced angiogenesis and tumor growth were observed [40]. These data indicate that both pro-inflammatory molecules are linked to tumor angiogenesis.

Altogether, our results demonstrate that kahweol is a potent anti-angiogenic compound both *in vitro* and *in vivo*, targeting some key steps shared with tumor progression, key molecules involved in ECM remodeling (MMP-2 and uPA), and key molecules involved in inflammation (COX-2 and MCP-1). All these effects may open a window for the potential therapeutical application of kahweol as an anti-angiogenic drug. In fact, recent epidemiological data show that coffee drinking diminishes the risk of some cancers [41], [42]. Furthermore, the inhibitory effects of kahweol on COX-2 and MCP-1 reinforce the idea of kahweol being a multi-targeted natural compound with high pharmacological potential. Further investigations with animal models seem warranted.

## Materials and Methods

### Ethics statement

All the manipulations of animals were carried out following the rules provided by the bioethical committee of the University of Málaga and permission according to RD1201/2005 provided by Consejería de Agricultura y Pesca (Andalusian Government). This study is part of a research project approved by the bioethical committee of the University of Málaga.

### Material and reagents

Cell culture media were purchased from Gibco (Grand Island, NY, USA) and Cambrex (Walkersville, MD, USA). Fetal bovine serum (FBS) was a product of Harlan-Seralab (Belton, U.K.). Matrigel was purchased from Becton Dickinson (Bedford, MA, USA), and Calcein-AM was from Molecular Probes (Eugene, OR, USA). Kahweol was supplied by Sigma-Aldrich (St. Louis, MO, USA). Stock solution (10 mg/mL) was prepared in DMSO and stored in aliquots at –20°C. In all the assays, the vehicle (DMSO) was at less than 1% (v/v) and controls with the vehicle alone were carried out in parallel. Supplements and other chemicals not listed in this section were obtained from Sigma-Aldrich. Plastic ware for cell culture was supplied by NUNC (Roskilde, Denmark).

### Cell cultures

Human umbilical vein endothelial cells (HUVEC) were isolated by a modified collagenase treatment, as previously reported [43], and maintained as we described elsewhere [10]. HT-1080 fibrosarcoma cells were supplied by ATCC and maintained in culture as described by the provider. Culture media for tumor cells were supplemented with 10% FBS, whereas culture medium for HUVEC was supplemented with 20% FBS, with the exception of the experiment under low proliferation rate conditions (2% FBS).

### *In vivo* angiogenesis CAM assay

The *in vivo* CAM assay was carried out as described elsewhere [21], using fertilised chick eggs, provided by Granja Santa Isabel (Córdoba, Spain). Briefly, eggs were incubated horizontally at 38°C in a humidified incubator, windowed by day 3 of incubation and processed by day 8. Kahweol stock solution was added to a 1% solution of methylcellulose in water, and 10 µL drops of this solution were allowed to dry on a Teflon-coated surface in a laminar flow hood. Then, the methylcellulose discs were implanted on the CAM, the eggs were sealed with adhesive tape and returned to the incubator for 48 h. Negative controls were always made with DMSO mixed with the methylcellulose. Six eggs were used for each tested dose of kahweol. After incubation, CAMs were examined under a stereomicroscope. The assay was scored as positive when two independent observers reported a significant reduction of vessels in the treated area.

### *In vivo* angiogenesis assay with fluorescent zebrafish

Zebrafish (*Danio rerio*) is being used as an easy *in vivo* study of angiogenesis and for the search of new modulators of angiogenesis [44]. Furthermore, the availability of transgenic lines of zebrafish exhibiting fluorescent blood vessels is allowing a rapid and precise analysis of vessels, thanks to the expression of a choral green fluorescent protein (G-RCFP) controlled by a promoter for VEGF-R2 [45].

One day after mating, eggs were transferred to a Petri dish, where they were treated with lye diluted to 0.5% in water for 90 s. Then, they were washed 3 times for 3 min with water, and maintained for 24 h at 28.5°C. After this new incubation, chorion was retired and larvae were transferred to 96-well plates (a larva per well) with 0.1 mL of water per well in the presence of the indicated concentration of kahweol. After an additional 24 h incubation at 28.5°C, effects on blood vessels were observed with a binocular lens with filters for fluorescence and photographs were taken from relevant images.

Video images of blood flow thru intersegmental vessels were taken on the caudal region next to vitellus in 48 h larvae after 24 h of treatment in the absence (control, Video S1) or presence (Video S2) of 50 µM kahweol.

### Detection of apoptosis in quail CAM

Apoptosis assays were carried out, after 24 h of incubation in the presence of kahweol, by staining of nuclei with Hoechst as described by us elsewhere [14].

### *Ex vivo* mouse aortic ring assay

C57BL/6 mice (six weeks in age) were sacrificed by isofluran inhalation according to the local ethics committee. Thoracic aorta was carefully dissected and aortic rings were cultured in 3D collagen gels, as previously described [46]. Cultures were maintained at 37°C under controlled humid atmosphere (5% CO_2_). The effects of kahweol, VEGF and the vehicle (DMSO) was tested by adding them to culture media at day zero. At different times of culture, rings were photographed under clear field illumination by using an inverted microscope with phase contrast Nikon Diaphot-TM (Nikon Corp., Tokyo, Japan). The angiogenic response was quantified by microvessel counting according to published criteria [47].

### *In vitro* angiogenesis assays

Different *in vitro* assays were carried out in order to test the specific effects of kahweol treatment on several key steps of the angiogenic process in both endothelial and tumor cells. First of all, the MTT cell proliferation assay was carried out to determine the long term (after three days of incubation) cytotoxicity of kahweol and to evaluate its IC_50_ value for endothelial cells, as described by us elsewhere [10]. Subsequently, additional *in vitro* angiogenesis assays were carried out both in the absence and presence of kahweol in the range of concentrations of its IC_50_ value in the MTT assay. These assays included apoptosis assay, tube formation by endothelial cells on Matrigel, zymographic assays for the detection of gelatinases and urokinase in conditioned media of control and kahweol-treated cells, cell migration “wound healing” assay, and fluorescent cell invasion assays. All of them are extensively described by us elsewhere [10], [12]. In all these *in vitro* assays, kahweol treatments were carried out under conditions (kahweol concentration and duration of treatment) that did produce no cytotoxic effect on cells. For zymographic assays, conditioned media and cell extracts were obtained as previously described by us [12]. The gelatinolytic assays were carried out in two different ways to obtain complementary information: firstly, cells were treated or not with the test compound and samples from these were submitted to gelatinase zymography to detect the effects of the kahweol treatment on the expression of gelatinase activities; secondly, in some experiments, samples from control, untreated HT-1080 fibrosarcoma cells were submitted to zymography and, after electrophoresis, different concentrations of kahweol were added to the substrate buffer to determine the potential direct effect of kahweol on gelatinase activity. In the invasion assay, fluorescence-labelled HUVEC cells are suspended in culture medium in the presence of different concentrations of kahweol and in the absence of serum into FluoroBlok inserts whose filters were coated with Matrigel. These inserts are added to wells containing complete culture medium with 10% FBS as chemoattractant in 24-well fluorescence opaque plates allowing only monitoring of fluorescence from the bottom.

### Expression of COX-2

Subconfluent HUVEC cultures were stimulated with PMA (50 ng/mL) for 4 h in the absence (controls) or presence of different concentrations of kahweol. After incubation, cells were washed twice with cold PBS and then lysed with cold lysis buffer (50 mM Tris, pH 7.4, 150 mM NaCl, 1% Tritox X-100, 0.25% sodium deoxycholate, 1 mM EDTA, 1 mM sodium orthovanadate and 5 mg/mL of a protease inhibitors mixture). Cells were scrapped, and maintained within a microfuge tube in ice for 15 min. Afterwards, extracts were centrifuged at 13,000 rpm for 15 min at 4°C. Supernatants were stored at −80°C until the moment of analysis. These samples were denatured for 5 min at 95°C and submitted to SDS-PAGE. After electrophoresis, samples were electro-transferred to nitrocellulose PROTRAN membranes, blocked with 5% dried skimmed milk in 50 mM Tris pH 8.4, 0.9% NaCl, 0.05% Tween 20 (Tris buffered saline-Tween 20, TBS-T), and incubated overnight in the presence of an anti-COX-2 (at a dilution of 1∶500) or anti-beta actin (at a dilution of 1∶3500) monoclonal antibodies (Santa Cruz Biotechnology and Sigma, respectively). After three washing steps with TBS-T, a horseradish peroxidase antibody (diluted 1∶10,000 in blocking buffer) was used as secondary antibody. After 1h of incubation at room temperature, samples were developed using the enhanced chemiluminescence system (GE Healthcare).

### Determination of secreted MCP-1

Conditioned media from untreated and kahweol treated HUVEC cells for 24 h were obtained. The secreted MCP-1 present in these conditioned media was quantified by using a MCP-1 human Biotrak Easy ELISA (GE Healthcare), following supplier's instructions.

### Statistics and image analysis

All quantitative data are expressed as means ± standard deviation (S.D.). Two-tailed Student's *t-*test was used for evaluations of pair of means, to establish which groups differed from the control group. Quantitative analysis of images was performed with the NIH Image 1.6 Program.

## Supporting Information

Video S1 **Blood flow thru intersegmental vessels in control, untreated 48 h zebrafish larvae.**
(AVI)

Video S2 **Blood flow thru intersegmental vessels in 48 h zebrafish larvae treated for 24 h with 50 µM kahweol.**
(AVI)
