# Correction: Anti-Angiogenic and Anti-Inflammatory Properties of Kahweol, a Coffee Diterpene

**DOI:** 10.1371/annotation/38262cc6-07cc-4074-8ce7-2181d4d0fbdc

**Published:** 2011-11-01

**Authors:** Casimiro Cárdenas, Ana R. Quesada, Miguel A. Medina

The correct Reference 45 is:

45. Lawson ND, Weinstein BM (2002) In vivo imaging of embryonic vascular development using transgenic zebrafish. Dev Biol 248:307–318. 

